# The Growth of Easements as a Conservation Tool

**DOI:** 10.1371/journal.pone.0004996

**Published:** 2009-03-26

**Authors:** Isla S. Fishburn, Peter Kareiva, Kevin J. Gaston, Paul R. Armsworth

**Affiliations:** 1 Biodiversity and Macroecology Group, Department of Animal and Plant Sciences, University of Sheffield, Sheffield, United Kingdom; 2 The Nature Conservancy, Santa Clara University Project Office, Santa Clara, California, United States of America; University of Kent, United Kingdom

## Abstract

**Background:**

The numerous studies examining where efforts to conserve biodiversity should be targeted are not matched by comparable research efforts addressing how conservation investments should be structured and what balance of conservation approaches works best in what contexts. An obvious starting point is to examine the past allocation of effort among conservation approaches and how this has evolved.

**Methodology/Principal Findings:**

We examine the past allocation of conservation investment between conservation easements and fee simple acquisitions using the largest land trust in operation, The Nature Conservancy (TNC), as a case study. We analyse the balance of investments across the whole of the US and in individual states when measured in terms of the area protected and upfront cost of protecting land.

**Conclusions/Significance:**

Across the US as a whole, the proportion of conservation investment allocated to easements is growing exponentially. Already 70% of the area of land protected in a given year, and half of all the financial investment in land conservation, is allocated to easements. The growth rate of conservation easements varies by a factor of two across states when measured in terms of the area protected and by a factor of three in terms of financial expenditure. Yet, we were unable to find consistent predictors that explained this variation. Our results underscore the urgency of implementing best practice guidelines for designing easements and of initiating a wider discussion of what balance of conservation approaches is desirable.

## Introduction

Habitat loss is the primary driver of terrestrial biodiversity declines [1]–[3]. Preventing further imperilment requires significant expansion of current land conservation efforts [4]. Yet, the extent to which land can be completely removed from economic production to allow for nature reserves in public or NGO (Non Governmental Organisation) ownership is limited [5]–[6]. Instead, conservation organisations increasingly rely upon voluntary methods to conserve biodiversity on private land alongside low impact uses [7]–[8]. What balance of conservation approaches is desirable, from more narrowly concentrated full protection through nature reserves to partial protection efforts that are more broadly distributed and coexist alongside low impact production systems, is a source of continuing debate within the conservation community [9]–[11].

The simplest approach to land conservation is through fee simple acquisition. Here, the conservation group/agency takes full ownership of the land. A fee simple approach is well-suited to areas that are subject to a threat that can only be prevented if the land is acquired outright, or when land is exposed to numerous threats that cannot be tackled in a piecemeal manner. While useful for protecting a few special areas of conservation interest, the upfront costs of acquiring land outright can be high when compared to other conservation approaches. In addition, management costs can also be higher because the conservation group acquiring the land either has to manage it themselves or find someone else to undertake this for them.

Conservation easements provide an alternative approach to land conservation. Easements first achieved prominence as a tool to protect habitat in the US, but are now being used in Latin America, Australia and the Pacific [12]–[14]. Easements involve voluntary legal contracts allowing lands to remain in private ownership, yet restrict the rights of the property owner in a specific way that fosters conservation [15], [16]. For example, large forest easements may prevent housing developments and require certified sustainable harvest practices. The landowner often receives financial compensation or tax breaks in return for the restriction on activities. Most easements are purchased and held by land trusts in the US. For example, local and state land trusts opted to make 60% of their investments in land conservation using easements in 2000 [17]. State, national and local governments may also hold easements, and often land trusts may obtain an easement and transfer it to a public agency. Easements clearly do not offer as secure protection for habitat as acquiring land outright. Yet, easements allow lands to remain productive and in private hands, which is often seen as politically desirable. Little research has examined actual conservation benefits provided by easements [18]–[20]. Acquisition costs of easements are thought to be generally lower than those of fee simple purchases. However, additional monitoring, defence and other transaction costs can result, because of the fragmentary property rights [15]. The cost effectiveness of easements at acquisition itself depends on the information available to the conservation group or agency about private landowners' valuations of their properties [21].

Resources for conservation are limited and must be allocated effectively [22]. To date, most studies exploring conservation allocation decisions have focused on what locations should be priorities for conservation [23]–[27]. An equally important yet neglected issue concerns what balance of conservation approaches is most effective and what conservation tool is best suited to given ecological, cultural and socioeconomic contexts. The example of fee simple acquisition and easements demonstrates how two approaches to land conservation can offer different advantages and be better suited to some contexts than others [15], [21]. Investment choice when dealing with a specific property or landowner may be restricted, because that individual may only be interested in one type of land deal. Across a region or a whole organization, however, strategic choices must be made about what balance of approaches the conservation group should have in its portfolio of protected sites.

When discussing what balance of conservation approaches is desirable, a starting point is to ask how conservation groups currently allocate their effort across different investment strategies and how that is changing. Here, we examine what determines the balance of conservation approaches, using The Nature Conservancy (TNC) as a case study. TNC is the world's largest land trust and by 2008 had protected over 17.2 million acres of habitat across the US at an upfront cost of over USD $7.5 billion. We explored what balance of approaches TNC has taken to protect land. Our study is unusual in examining the balance of conservation efforts both using the area protected and the financial cost of that investment. We first examined how the proportion of investment allocated to easements has changed through time across the whole of the US. However, a striking feature of easement versus land acquisition is that the rate of growth in easement usage varies substantially across states. Therefore, we then asked what factors explain variation among states in the rate at which easements are being adopted. Since the spread of easements is relatively recent compared to the use of nature reserves or land acquisition, our analysis is in some sense a study in the early adoption of a new approach to conservation.

## Methods

### Data set

We analysed all conservation easement and fee simple transactions made by TNC in the 48 contiguous states between 1954 and 2003. Details of how TNC operate are described in Fishburn et al. [27]. Throughout the paper, we use the proportion of the overall investment in land conservation that was made using easements 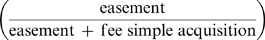 to summarise the allocation decision between the two conservation approaches. We analyzed how this proportion has changed through time across the whole of the US and for individual states. Currently, the full extent of the TNC dataset only permits analyses at or above the state level. States provide a meaningful grain for the analysis, because variations in state tax codes and land management practices will influence the choice of conservation strategies; indeed, in a companion paper we evidence how biological and socioeconomic factors combine to determine state-level variation in overall investment patterns within these data [27].

For all analyses, we measured conservation effort both by the total area of land protected and upfront cost of achieving that protection. We did not have data regarding ongoing management costs. All dollar values were converted to 2003 equivalents using the Consumer Pricing Index [28] to account for inflation. Properties that were fully donated to TNC only appear in the area tallies; partially donated deals (i.e. those acquired by TNC at a fraction of their fair market value) appear in both tallies.

There were enough land transactions for us to use the proportion of investments made via easements in each year when analyzing the allocation decision between the two conservation approaches across the whole of the US. When moving to the individual state level, we pooled the data into two time periods to maintain adequate sample sizes (Text S1; Table S1). Some states still did not have sufficient transactions and these were omitted from the state level analyses (leaving 44 states for the area measure and 40 states for cost). The proportion of investments made using easements typically grew between the two time periods, and we computed and analysed the annual growth rate for each state.

### Predictor variables

We selected six biological and socioeconomic variables that could potentially explain state level differences in the allocation decision between easements and fee simple acquisitions: species richness, the area of the state, the proportion of the state that is in agricultural uses, the average price of agricultural land, the threat of development as approximated by the rate of change in number of households; and the proportion of land protected by other land trusts using easements versus a fee simple approach (29–32; Table 1).

**Table 1:** Predictor variables tested to explain where The Nature Conservancy allocates conservation investments.

Predictor	Source	Additional comments	Transformation^a^
State area (acres)	US Census Bureau (2000)		i. Box–Cox (λ = 0.43)ii. Box–Cox (λ = 0.42)
Species richness	NatureServe (2006)	National distribution of all native terrestrial vertebrates, invertebrates and plants	i. Log transformationii. Log transformation
Cost	US Census of Agriculture (2002)	Average land market value between 1974 to 2002; proxy for land cost	i. Log transformationii. Log transformation
Households	US Census Bureau (2000)	Rate of change in number of households between 1960–2000; proxy for land threat	i. Log transformationii. Box–Cox (λ = 0.03)
Farms	US Census Bureau (2000)	Proportion of the average land occupied as farmland between 1974 to 2002	i. Arcsine square rootii. Arcsine square root
LTA easements	Land Trust Alliance (2003)	Proportion land protected as easements by other land trusts	i. Arcsine square rootii. Arcsine square root

a Differences in sample size between the two response variables meant that, in some circumstances, the same transformation did not allow for the data to meet assumptions. Therefore, two transformations were applied to each predictor. Transformations correspond with the response variables exploring the annual growth rate of investments that are easements for (i) acres and (ii) dollars. The first transformation was that used for acres; the second was that used for dollars.

### Statistical Analyses

To meet the assumptions of normality transformations were performed (Table 1). Where appropriate, predictor variables were log or arcsine square root (that deals with proportion data) transformed. When this was not suitable a more flexible Box–Cox transformation was applied [33]. The response variable, annual growth rate of easement acres was log transformed. A Box–Cox transformation was applied to the response variable, annual growth rate of easement dollars. All analyses were performed using the transformed data.

We used non-spatial modelling techniques. To test the appropriateness of these methods, spatial dependency was checked in the response variables. We examined correlations between adjoining pairs of states (one lag only). Bootstrapping revealed no significant correlations. Furthermore, the observed correlations themselves explained little variation (r^2^<0.01 for both acres and dollars).

Our analyses explore the variation in the two response variables (growth rate in the proportion of easement acres and growth rate in the proportion of easement dollars) across the US as a whole and across individual states. For the whole US dataset, we used regressions to examine temporal trends in the proportion of investments made with easements between 1954 to 2003.

To explore variations in the allocation between the two response variables across different states, we regressed the growth rate in the proportion of easements in each state as measured by acres and dollars against each of the predictor variables individually (Table 1). Quadratic terms were also included in these bivariate regressions to check for non-linear relationships. We used Akaike's Information Criterion (AIC; 34) to compare linear and quadratic regressions. The most parsimonious model (i.e. model with lowest AIC) was selected.

To explore variables in combination, we then performed multiple regressions adopting an information theoretic approach. We constructed models for all possible combinations of predictor variables. Because the most parsimonious models in the bivariate regressions only involved linear terms, we omitted quadratic terms from this multiple regression analysis. We also omitted interaction terms. For each model we calculated its model weight and used AIC [34] to identify the most parsimonious model. Following Johnson & Omland [34], we then constructed the 95% confidence set of models, i.e. the smallest number of models whose cumulative weights summed to 0.95. Before running multiple regressions, we checked for collinearity among the predictor variables [34]. In all cases tolerance levels were sufficiently high (i.e. >0.1).

## Results

### Whole US

The Nature Conservancy had protected over 3.1 million acres of habitat using easements (an area comparable to that of Connecticut) at an upfront cost of USD $0.92 billion between 1954 and 2003. A further 5.3 million acres of land (an area larger than Massachusetts) was protected at a cost of USD $4.8 billion using a fee simple approach.

**Figure 1:**
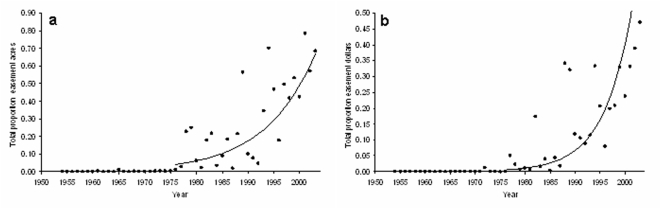
Temporal growth of the proportion of overall conservation effort allocated to easements for all states combined. Proportion allocated to easements is measured as (a) acres protected and (b) dollars invested across the coterminous states. The log of these proportions were regressed against time for the period of peak easement activity, 1976–2003. (A) Acres: y = −208+0.10*t*; n = 27; p<0.0001; r^2^ = 0.48 and (B) Dollars: y = −360+0.18*t*; n = 27; p<0.0001; r^2^ = 0.54. When graphed on normal axes these fits produced the exponential curves shown.

Easements began to be employed widely in the 1970s (Fig. 1). Since then the proportion of overall conservation effort allocated to easements has been growing steadily and there is no evidence of this process saturating. There was a highly significant positive relationship between year and the proportion of investments made as easements measured both in acres and in dollars (Fig. 1; r^2^ = 0.64, p<0.0001; r^2^ = 0.54, p<0.0001, respectively). By 2003, 70% of the area protected was via conservation easements and nearly half of all of the financial expenditure went on easements.

The year-on-year change in how important easements are becoming is more pronounced when viewed in financial terms than just in terms of area. On comparing the slopes from the two regression models, the slope measuring the growth of the easement proportion in dollars was steeper than that measured in acres, but significance was marginal (F = 3.87, df = 1,52, p = 0.05).

### State level

**Figure 2:**
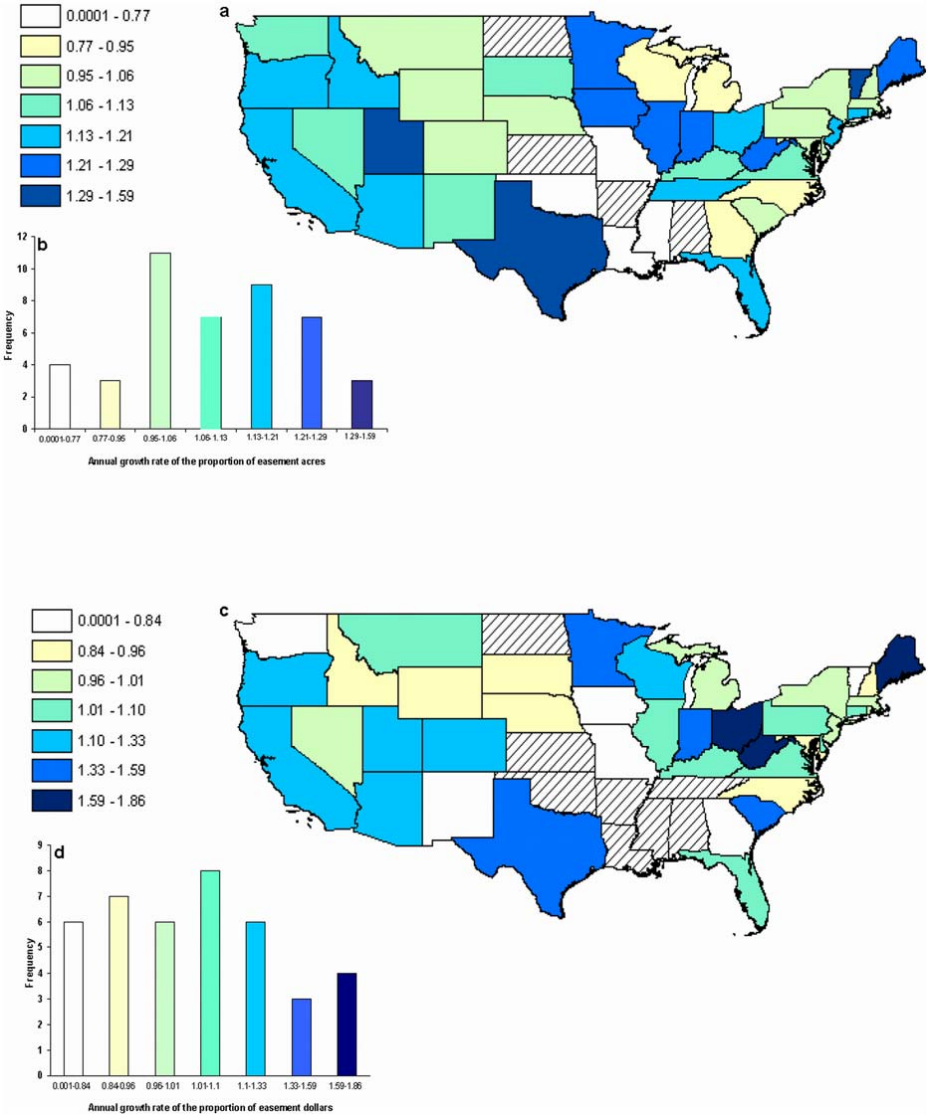
Spatial distribution of annual growth rate in the proportion of easements (a) acres protected and (c) dollars invested by The Nature Conservancy across the US. (b) Frequency distribution of annual growth rate in the proportion of acres protected and (d) dollars invested. For (a) and (b) data was over a 42 year period, ranging from 1961–2003; for (c) and (d) data was over a 31 year period, ranging from 1972–2003. Hatched states were not included.

**Figure 3:**
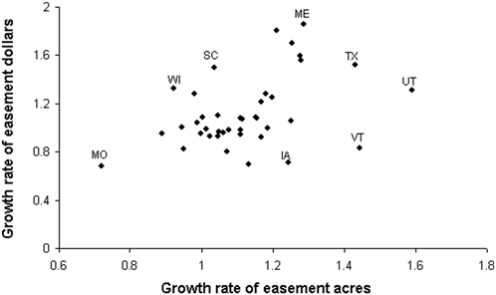
Annual growth rate in the proportion of overall conservation effort allocated to easements. Growth rate measured as area protected and dollars invested for each year across 40 states (r^2^ = 0.42; n = 40; p<0.01; for the transformed data). Regardless of how conservation effort was measured, the growth in importance of easements was fast in some states (e.g. TX, Texas; ME, Maine; UT, Utah). Others had a high annual growth rate when conservation was measured in terms of area (e.g. VT, Vermont; IA, Iowa) or dollars (e.g. WI, Wisconsin; SC, South Carolina). The rate of uptake of easements increased slowly in Missouri (MO) by either measure of conservation effort.

Easements are growing in importance relative to the overall investment profile at different rates across the US (Fig. 2). The spatial patterns in the rates of growth of easements when measured by area or financial outlay are positively correlated (Fig. 3; r^2^ = 0.42, p<0.01). However, much of the variance remains unexplained, suggesting that the two measures of conservation effort are complementary. The rate of growth of easements was rapid no matter how it was measured in some states (e.g. TX, Texas; UT, Utah; ME, Maine; Fig. 3). In others, easements appeared to be growing in importance faster when conservation effort was measured by area than when measured by financial investment (e.g. VT, Vermont; IA, Iowa; Fig. 3), and vice versa (e.g. WI, Wisconsin; SC, South Carolina; Fig. 3). Missouri had a decrease in the rate of uptake of easements in both measures of investment.

### Bivariate

For bivariate regressions, the six independent hypotheses (Table 1) were rejected when considering the rate of uptake of easements on an area basis. For financial expenditure, there was a significant positive relationship between the annual growth rate of the proportion of TNC easements and the proportion of land protected with easements by other land trusts, but little of the variation could be explained (r^2^ = 0.10; p<0.05).

### Multivariate

In multiple regressions there were no significant predictors explaining state level variation in the rate of uptake of easements whether measured by area or upfront cost. In contrast, a small set of predictors proved relatively successful at explaining state level variation in the overall amount of investment in easements and fee simple purchases in a companion paper [26].

## Discussion

To help understand how best to distribute conservation efforts, we analysed the allocation of resources between different investment strategies. We focused our study on the growth of easement deals as a proportion of total investments across the whole of the US by the largest land trust, TNC. We also analysed the partitioning of investments across selected states using both area protected and upfront cost as investment metrics. Similar more localized analyses at finer spatial resolution would also be worthwhile.

The proportion of investments made as easements continues to grow exponentially. This growth by area is reflected elsewhere in the land trust movement. Easement protection by local and state land trusts rose from 2.5 million acres in 2000 to over 6.2 million acres in 2005 [17]. Easements in the US were first used as a method of protection in the late 1880s [15], [36]. Land trusts have used them to protect property since the late 1950s [37], with their popularity only really increasing in the 1970s [26]. For TNC, easements began to see widespread uptake around 1976. This is the year the Tax Reform Act granted conservation easements a reduction from federal income tax [38]. Federal and state tax incentives are suggested to have contributed to the growth of conservation easements among land trusts across the US [13]. The rapid growth of easements emphasizes the urgency of developing best practice guidelines for the establishment and monitoring of easements to ensure effective conservation designs.

While the growth rate of easements on an area basis has been noted previously [18], our paper provides the first demonstration that this growth reflects the overall allocation of conservation funds to acquire properties and is not just a function of easement donations. In the TNC dataset, properties that were fully donated to TNC (i.e. acquisition cost = USD $0.00) accounted for over 645 thousand acres of habitat or 12% of the total area protected as fee simple. Full donations of easements accounted for over 785 thousand acres or 25% of the total easement area. Partial donations also occur in which TNC purchase properties under fee simple or easement arrangements for less than their fair market value, but we are unable to identify these from within the current dataset.

Given their later uptake, one would expect the allocation of conservation effort to easement deals to increase with time, but at some point this growth must stabilize. Hopefully, stabilization will occur near some optimum balance between the two investment approaches, reflecting the different advantages they offer as conservation tools. We would encourage wider discussion of what determines the optimal balance for biodiversity conservation of narrow and focused investments, like fee simple acquisitions, to broader shallower investments, as offered by easements. Any such discussion must recognise, however, that the outcome will be context dependent and will vary across conservation goals and organizations.

Exploring the balance of conservation approaches across the whole US conceals the pattern of growth across states. There was large state level variation in the annual growth rate of investments. Many of the states with slow growth rates were in the central and southern US. This was even more apparent when considering states that we excluded from our analyses precisely because they had too few easement deals.

While we find spatial differences in the growth rate of easements, we were unable to identify consistent predictors explaining this allocation pattern. In marked contrast, we have shown elsewhere that a relatively small set of biological and socioeconomic factors are good predictors of the allocation of overall conservation efforts across states by TNC [27]. For example, 53% of total area protected by TNC across US states was explained by species richness, land price, rate of development, the activity of other land trusts and state area, although state area contributed very little to the variance explained. Similar predictors (except state area) explained 52% of the variation in TNC's financial expenditure. This contrast is indicative of the wider discussions in conservation biology where a great deal has been written about where investments should be directed [23], [25], but almost nothing has been written about how we should structure conservation investments and what type and combination of conservation approaches works best in different ecological, cultural and socioeconomic contexts [21]. Our results suggest that past allocation of resources to the two conservation approaches perhaps owes more to the particular staff in TNC's state chapters and their individual experiences than it does to a systematic decision making process.

## Supporting Information

Text S1 (0.02 MB DOC)

Table S1 Supplementary table corresponding to manuscript
(0.11 MB DOC)
