# Supplementary material for: The Growth of Easements as a Conservation Tool
Source: PLoS One. 2009 Mar 26;4(3):e4996. doi: 10.1371/journal.pone.0004996 (PMC2659785; doi:10.1371/journal.pone.0004996)
Supplement: Text S1 — (0.02 MB DOC) [file pone.0004996.s001.doc]

**On-line supplementary material**

The date of the first and last easement deal varied across individual states. For each state, investment totals were summed across two bins of equal duration (Time Period 1 and Time Period 2) and attributed to the mid-point of each bin. An annual growth rate was then calculated from the growth in area or financial investment that occurred between these two time points. The relevant time periods for each state are reported (Table S1).
