# Supplementary material for: The Growth of Easements as a Conservation Tool
Source: PLoS One. 2009 Mar 26;4(3):e4996. doi: 10.1371/journal.pone.0004996 (PMC2659785; doi:10.1371/journal.pone.0004996)
Supplement: Table S1 — Supplementary table corresponding to manuscript (0.11 MB DOC) [file pone.0004996.s002.doc]

**On-line supplementary material**

The date of the first and last easement deal varied across individual states. For each state, investment totals were summed across two bins of equal duration (Time Period 1 and Time Period 2) and attributed to the mid-point of each bin. An annual growth rate was then calculated from the growth in area or financial investment that occurred between these two time points. The relevant time periods for each state are reported in the table below.

Table S1. Time periods selected to calculate the annual growth rate in the proportion of investments that are easements across states.

| State | Acres | | Dollars | |
| --- | --- | --- | --- | --- |
|  | TP_1_ | TP_2_ | TP_1_ | TP_2_ |
| Alabama | Excluded | | Excluded | |
| Arkansas | Excluded | | Excluded | |
| Arizona | 11/07/83 to  26/08/93 | 27/08/93 to  13/10/03 | 16/04/90 to  13/01/97 | 14/01/97 to 13/10/03 |
| California | 10/05/72 to  28/02/88 | 29/02/08 to  19/12/03 | 10/05/72 to 28/02/88 | 01/03/88 to  19/12/03 |
| Colorado | 17/11/96 to  03/06/90 | 04/06/90  to 19/12/03 | 04/02/82 to 24/12/92 | 25/12/92 to  13/11/03 |
| Connecticut | 15/07/61 to 27/08/82 | 28/08/02 to  10/10/2003 | 05/04/88 to 07/01/96 | 08/01/96 to 10/10/03 |
| Delaware | 28/06/1993  to 28/09/98 | 29/09/98 to 30/12/03 | 28/06/93 to 28/09/98 | 29/09/98 to 30/12/03 |
| Florida | 19/07/78 to 10/11/90 | 11/11/90 to 05/03/03 | 22/12/92 to 27/01/98 | 28/01/98 to 05/03/03 |

| State | Acres | | Dollars | |
| --- | --- | --- | --- | --- |
|  | TP_1_ | TP_2_ | TP_1_ | TP_2_ |
| Georgia | 31/05/83 to 10/09/83 | 11/09/83 to 22/12/03 | 31/05/83 to 07/03/93 | 08/03/93 to  13/12/02 |
| Iowa | 02/11/82 to 17/05/83 | 18/05/83 to 31/12/03 | 06/02/01 to 05/07/02 | 06/07/02 to 01/12/03 |
| Idaho | 09/04/81 to 19/08/92 | 20/08/92 to 31/12/03 | 09/04/81 to 13/08/92 | 14/08/92 to 18/12/92 |
| Illinois | 20/12/77 to 08/09/1990 | 09/09/90 to 28/05/03 | 08/04/99 to 02/05/01 | 03/05/01 to 28/05/03 |
| Indiana | 07/08/76 to 04/05/90 | 05/05/90 to 29/12/03 | 07/09/76 to 04/05/90 | 05/05/90 to  29/12/03 |
| Kansas | Excluded | | Excluded | |
| Kentucky | 05/02/97 to 29/05/99 | 30/05/99 to 24/06/02 | 05/02/97 to 15/07/00 | 16/07/00 to 23/12/03 |
| Louisiana | 03/05/96 to 29/05/99 | 30/05/99 to 24/06/02 | Excluded | |
| Massachusetts | 19/12/78 to 21/06/91 | 22/06/91 to 23/12/03 | 02/02/88 to  16/12/95 | 17/12/95 to 29/10/03 |
| Maryland | 15/09/75 to 06/02/89 | 07/02/89 to 02/07/02 | 12/12/77 to  23/03/90 | 24/03/90 to 02/07/02 |
| Maine | 21/12/73 to 21/12/88 | 22/12/88 to 23/12/03 | 03/06/87 to 12/09/95 | 13/09/95 to 23/12/03 |
| Michigan | 20/12/96 to 19/06/2000 | 20/06/2000 to 18/12/03 | 20/12/96 to 15/06/00 | 16/06/00 to 11/12/03 |
| Minnesota | 24/06/82 to 27/12/92 | 28/12/92 to 03/07/03 | 24/06/82 to 27/12/92 | 28/12/92 to 03/07/03 |
| Missouri | 18/04/85 to  20/09/98 | 21/09/98 to 22/02/02 | 18/04/95 to 20/09/98 | 21/09/98 to 22/02/02 |

| State | Acres | | Dollars | |
| --- | --- | --- | --- | --- |
|  | TP_1_ | TP_2_ | TP_1_ | TP_2_ |
| Mississippi | 09/01/92 to 06/07/95 | 07/07/95 to 31/12/98 | Excluded | |
| Montana | 26/04/76 to 09/01/90 | 10/01/90 to 25/09/03 | 23/04/80 to 01/11/91 | 02/11/91 to 12/05/03 |
| North Carolina | 24/12/86 to 21/06/95 | 22/06/95 to 17/12/03 | 31/08/84 to 16/09/92 | 17/09/92 to 03/10/00 |
| North Dakota | Excluded | | Excluded | |
| Nebraska | 23/09/82 to 12/05/93 | 13/05/93 to  30/12/03 | 23/09/82 to 12/05/93 | 13/05/93 to 30/12/03 |
| New Hampshire | 15/03/74 to 29/01/89 | 30/01/89 to 16/12/03 | 09/06/86 to 13/03/95 | 14/03/95 to 16/12/03 |
| New Jersey | 28/10/77 to 29/11/90 | 30/11/90 to 31/12/03 | 19/12/86 to 25/06/95 | 26/06/95 to 31/12/03 |
| New Mexico | 20/12/84 to 22/06/94 | 23/06/94 to 24/12/03 | 01/06/94 to 09/12/98 | 10/12/98 to 19/06/03 |
| Nevada | 14/09/89 to 13/03/96 | 14/03/96 to 10/09/02 | 14/09/89 to 14/01/96 | 15/01/96 to 15/05/02 |
| New York | 27/11/68 to 31/05/86 | 1/06/86 to 03/12/03 | 11/03/85 to 22/07/94 | 22/07/94 to 03/12/03 |
| Ohio | 22/12/80 to 29/04/92 | 30/04/92 to 05/11/03 | 22/12/02 to 20/12/91 | 21/12/91 to 17/12/02 |
| Oklahoma | 17/09/85 to 08/10/92 | 09/10/92 to 29/03/89 | Excluded | |
| Oregon | 07/09/76 to 16/12/89 | 17/12/89 to 27/03/03 | 15/02/79 to 07/03/91 | 08/03/91 to 27/03/03 |
| Pennsylvania | 26/12/74 to 05/02/89 | 06/02/89 to 19/03/03 | 30/11/98 to 23/01/01 | 24/01/01 to 19/03/03 |

| State | Acres | | Dollars | |
| --- | --- | --- | --- | --- |
|  | TP_1_ | TP_2_ | TP_1_ | TP_2_ |
| Rhode Island | 13/10/72 to 21/05/88 | 22/05/88 to 29/12/03 | 23/12/82 to 26/06/93 | 27/06/93 to 29/12/03 |
| South Dakota | 07/04/87 to 17/02/95 | 18/02/95 to 31/12/02 | 07/04/87 to 09/08/94 | 10/08/94 to 11/12/01 |
| Tennessee | 22/11/82 to 22/01/93 | 23/01/93 to 25/03/03 | Excluded | |
| Texas | 31/12/83 to 23/12/93 | 24/12/93 to 17/12/03 | 29/09/86 to 21/11/94 | 22/11/94 to 13/01/03 |
| Utah | 22/12/94 to 16/06/99 | 17/06/99 to 10/12/03 | 22/12/94 to 17/12/98 | 18/12/98 to 13/12/02 |
| Virginia | 28/08/85 to 28/10/94 | 29/10/94 to 29/12/03 | 31/12/86 to 25/06/95 | 26/06/95 to 19/12/03 |
| Vermont | 30/10/75 to 30/09/89 | 01/10/89 to 02/09/03 | 30/11/90 to 09/09/96 | 10/09/96 to 20/06/02 |
| Washington | 08/12/75 to 29/07/89 | 30/07/89 to 21/03/03 | 14/01/99 to 15/02/01 | 16/02/01 to 21/03/03 |
| Wisconsin | 07/12/76 to 15/06/90 | 16/06/90 to 22/12/03 | 08/10/85 to 21/10/94 | 22/10/94 to 03/11/03 |
| West Virginia | 31/12/84 to 31/12/91 | 01/01/92 to 30/12/98 | 30/10/89 to 30/11/93 | 01/12/93 to 31/12/97 |
| Wyoming | 17/12/76 to 18/06/90 | 19/06/90 to 19/12/03 | 05/03/91 to 23/07/97 | 24/07/97 to 12/12/03 |

TP_1_ = time period 1

TP_2_ = time period 2
